# Supplementary material for: Drug repurposing for aging research using model organisms
Source: Aging Cell. 2017 Jun 16;16(5):1006–15. doi: 10.1111/acel.12626 (PMC5595691; doi:10.1111/acel.12626)
Supplement: Supplementary file 7 — Data S1 Zip‐Archive of all report cards. [file ACEL-16-1006-s007.zip › RC_MYR.pdf]

MYR

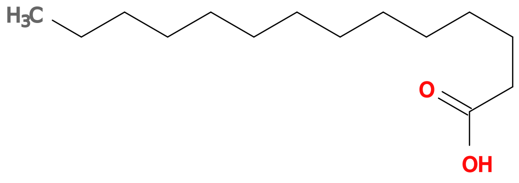

Database identifiers

ChEMBLCompound	CHEMBL111077
DrugBank	DB08231
CHEBI	28875
eMolecules	480439

Ranking

	Rank	Score
Drosophila	258/697	0.638
C. elegans	122/591	0.302

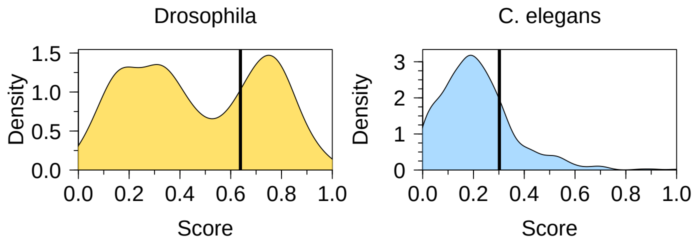

	Ageing implication	Domain conservation	Binding site conservation	Binding affinity	Bioavailability	Lipinski	Promiscuity	Purchasability	Drug approval	**Total**
Drosophila	1.0	0.923	0.913	0.612	(0.9)	0.0	-0.001	0.1	0.075	0.638
C. elegans	1.0	0.918	0.939	0.612	0.243	0.0	-0.001	0.1	0.075	0.302

Names

- **myristic acid**
- 1-tetradecanecarboxylic acid
- 14
- 14:0
- 14:00
- acide tetradecanoique
- C14
- Ch3-ch212-cooh
- Myristinsaeure
- tetradecoic acid

Roles

Daphnia magna metabolite, algal metabolite, EC 3.1.1.1 (carboxylesterase) inhibitor, human metabolite

Status

Approved drug (according to ChEMBL)	No
Classification (according to DrugBank)	experimental
Number of Rule of 5 violations	0
Binding affinity to original target in log units (RF-Score prediction)	5.46
Burns *C. elegans* bioavailability prediction	-2.43

Compound Target Characteristics

Tyrosine-protein kinase ABL1

Best gene implication in ageing for this target family came from gene P00519 via mapping the annotation from Ensembl ENSG00000097007 via mapping the annotation from EntrezGene 25 via mapping the annotation from GenAgeHuman 0078 annotated in GenAge release 17.

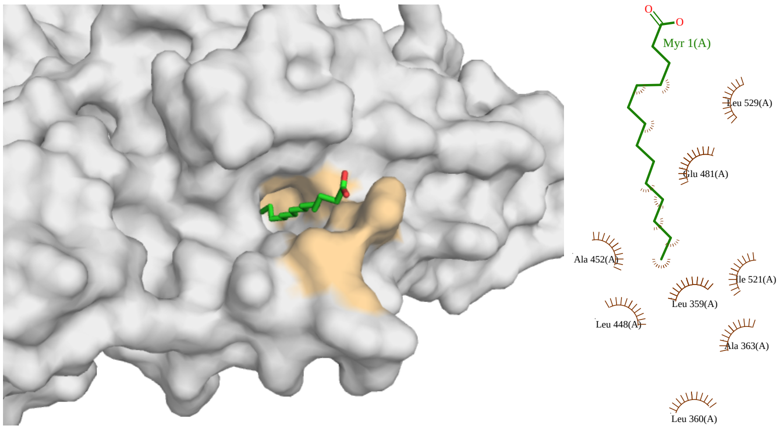

protein	amino acids contacts (binding site)
PDB:1opj:chainA:P00520	L L A L A E G I V L
PDB:1opk:chainA:P00520	L L A L A E G I V L
sp:P00519:ABL1_HUMAN	L L A L A E G I V L
tr:Q59FK4:Q59FK4_HUMAN	L L A L A E G I V L
tr:F1M0A6:F1M0A6_RAT	L L A L A E G I V L
tr:D3ZGM3:D3ZGM3_RAT	L L A L A E G I V L
tr:E9PT20:E9PT20_RAT	L L A L A E G I V L
tr:Q3V2W1:Q3V2W1_MOUSE	L L A L A E G I V L
sp:P00520:ABL1_MOUSE	L L A L A E G I V L
tr:Q3SYK5:Q3SYK5_MOUSE	L L A L A E G I V L
sp:P00522:ABL_DROME	L L A L A P G I V L
tr:A8JNU2:A8JNU2_DROME	L L A L A P G I V L
sp:P03949:ABL1_CAEEL	L V A L A Q G L V L

protein	whole protein ident	simil	domain-based ident	simil	contact-based ident	simil
PDB:1opj:chainA:P00520	0.99	1.0	1.0	1.0	1.0	1.0
PDB:1opk:chainA:P00520	1.0	1.0	1.0	1.0	1.0	1.0
sp:P00519:ABL1_HUMAN	0.88	0.95	0.93	0.98	1.0	1.0
tr:Q59FK4:Q59FK4_HUMAN	0.83	0.92	0.93	0.98	1.0	1.0
tr:F1M0A6:F1M0A6_RAT	0.93	0.96	0.97	0.99	1.0	1.0
tr:D3ZGM3:D3ZGM3_RAT	0.96	0.99	0.97	0.99	1.0	1.0
tr:E9PT20:E9PT20_RAT	0.93	0.96	0.97	0.99	1.0	1.0
tr:Q3V2W1:Q3V2W1_MOUSE	0.33	0.33	1.0	1.0	1.0	1.0
sp:P00520:ABL1_MOUSE	1.0	1.0	1.0	1.0	1.0	1.0
tr:Q3SYK5:Q3SYK5_MOUSE	0.96	0.98	1.0	1.0	1.0	1.0
sp:P00522:ABL_DROME	0.29	0.51	0.65	0.85	0.9	0.91
tr:A8JNU2:A8JNU2_DROME	0.29	0.5	0.65	0.85	0.9	0.91
sp:P03949:ABL1_CAEEL	0.22	0.43	0.55	0.84	0.7	0.94

Abl (FBgn0000017) associated phenotypes

cell migration defective, cell shape defective, germline clone, heat sensitive, lethal - all die before end of pupal stage, maternal effect, mitotic cell cycle defective, neuroanatomy defective, neurophysiology defective, partially lethal - majority die, partially lethal - majority live, reduced, rescuable maternal effect, size defective, somatic clone, some die during pupal stage

(Information from FlyBase)

Abl (UniProt:P00522) annotation

Function: Arm and Abl proteins function cooperatively at adherens junctions in both the CNS and epidermis; critical for embryonic epithelial morphogenesis regulating cell shape changes and cell migration. Plays a critical role in transducing embryonic midline repulsive cues; may regulate cytoskeletal dynamics underlying a growth cone's response to midline cues. The ability of pCC/MP2 axons to correctly interpret midline repulsive cues and stay on the ipsilateral side is dependent on the strength of both Slit/robo and Abl-dependent signaling pathways. (PubMed:11756472, PubMed:12973825, PubMed:9635189).
Subcellular location: Cytoplasm.
Tissue specificity: Arm and ena colocalize with Abl at adherens junctions throughout development. (PubMed:11756472, PubMed:9635189).
Developmental stage: Expressed both maternally and zygotically. (PubMed:2832740).
Disruption phenotype: Both loss- and gain-of-function mutants exhibit neurons within the pCC/MP2 pathway that incorrectly project across the midline. Loss of Abl disrupts cell migration and cell shape changes during dorsal closure. (PubMed:11756472, PubMed:9635189).

(Information from UniProt)

abl-1 (WBGene00000018) associated phenotypes

apoptosis variant, gamma ray induced apoptosis increased, lipid synthesis increased, protein subcellular localization variant

(Information from WormBase)

Mitochondrial peptide methionine sulfoxide reductase

Best gene implication in ageing for this target family came from gene P08761 annotated in UniProt release 2014_02. Annotation GO 8340 (determination of adult lifespan) was Inferred from Mutant Phenotype

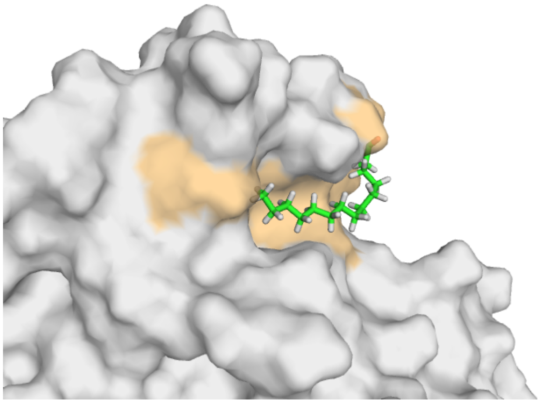

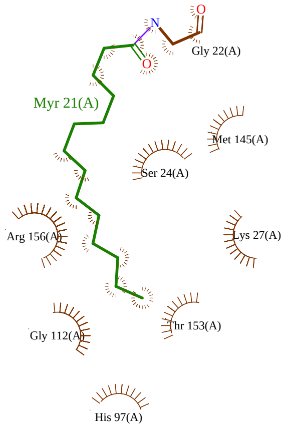

protein	amino acids contacts (binding site)							
PDB:2I9O:chainA:Q9D6Y7	G	S	K	H	G	M	T	R
tr:E5RIA9:E5RIA9_HUMAN	G	S	N	Y	G	M	T	R
sp:Q9UJ68:MSRA_HUMAN	G	S	N	Y	G	M	T	R
tr:F1LSJ6:F1LSJ6_RAT	G	S	K	Y	G	M	T	R
tr:M0RCC8:M0RCC8_RAT	G	S	K	Y	G	M	T	R
sp:Q923M1:MSRA_RAT	G	S	K	Y	G	M	T	R
sp:Q9D6Y7:MSRA_MOUSE	G	S	K	H	G	M	T	R
tr:Q7KUM8:Q7KUM8_DROME	T	R	-	S	D	I	-	A
sp:P08761:MSRA_DROME	T	R	-	S	D	I	-	A
tr:F2FB74:F2FB74_DROME	T	R	-	S	D	I	-	A
tr:B5X0L2:B5X0L2_DROME	T	R	-	S	D	I	-	A
tr:Q6NLA4:Q6NLA4_DROME	T	R	-	S	D	I	-	A
tr:O02089:O02089_CAEEL	-	-	-	K	D	R	-	Q
sp:P40029:MSRA_YEAST	-	-	-	E	D	N	T	R

	whole protein		domain-based		contact-based	
protein	ident	simil	ident	simil	ident	simil
PDB:2I9O:chainA:Q9D6Y7	1.0	1.0	1.0	1.0	1.0	1.0
tr:E5RIA9:E5RIA9_HUMAN	0.66	0.8	0.7	0.85	0.75	0.79
sp:Q9UJ68:MSRA_HUMAN	0.86	0.95	0.86	0.95	0.75	0.79
tr:F1LSJ6:F1LSJ6_RAT	0.72	0.75	0.76	0.8	0.88	0.9
tr:M0RCC8:M0RCC8_RAT	0.64	0.67	0.67	0.7	0.88	0.9
sp:Q923M1:MSRA_RAT	0.94	0.98	0.94	0.98	0.88	0.9
sp:Q9D6Y7:MSRA_MOUSE	1.0	1.0	1.0	1.0	1.0	1.0
tr:Q7KUM8:Q7KUM8_DROME	0.21	0.47	0.27	0.57	0.0	0.24
sp:P08761:MSRA_DROME	0.21	0.47	0.28	0.58	0.0	0.24
tr:F2FB74:F2FB74_DROME	0.21	0.48	0.28	0.58	0.0	0.24
tr:B5X0L2:B5X0L2_DROME	0.21	0.47	0.28	0.58	0.0	0.24
tr:Q6NLA4:Q6NLA4_DROME	0.21	0.47	0.28	0.58	0.0	0.24
tr:O02089:O02089_CAEEL	0.19	0.42	0.24	0.52	0.0	0.0
sp:P40029:MSRA_YEAST	0.26	0.55	0.29	0.57	0.25	0.08

Eip71CD (FBgn0000565) associated phenotypes

RU486 conditional, increased cell number, long lived, oxidative stress response defective, size defective

(Information from FlyBase)

Eip71CD (UniProt:P08761) annotation

Function: Has an important function as a repair enzyme for proteins that have been inactivated by oxidation. Catalyzes the reversible oxidation-reduction of methionine sulfoxide in proteins to methionine. (PubMed:12145281).
Induction: By ecdysone.

(Information from UniProt)
